# Supplementary material for: Alcohol Recognition and Desire to Drink of Extended Alcohol Brand Logos
Source: Int J Environ Res Public Health. 2022 Sep 17;19(18):11756. doi: 10.3390/ijerph191811756 (PMC9517033; doi:10.3390/ijerph191811756)
Supplement: Supplementary file 1 [file ijerph-19-11756-s001.zip › ijerph-1880807-supplementary.pdf]

Supplementary Materials
Questionnaire S1

(The original version was written in Thai language.)

Participant Information Sheet

1. I have read and understood the participant information statement. I agree to participate in the research project voluntarily.
    a. Yes, I agree.
    b. No, I disagree. (The end of data collection)

Section 1: Drinking behavior and alcohol-related products and media perception

1. Do you desire to drink alcohol at the moment?
    a. Yes, I do.
    b. No, I don't.
2. In the past 3 months, how often do you receive information from media, including social media?
    a. Everyday (7 days per week)
    b. Almost every day (5-6 days per week)
    c. Every other day (3-4 days per week)
    d. Every week (1-2 days per week)
    e. Every month (5-6 days per month)
    f. Once in a while (Less than once a month)
3. Which channels do you use to receive information in the past 3 months?
    a. Television
    b. Radio
    c. Newspaper
    d. Outdoor media (Poster, banner, outdoor billboards)
    e. Facebook
    f. Line application
    g. Instagram
    h. Twitter
    i. YouTube
    j. Website
    k. Others (please specify)

Section 2: Reaction time

In this section, the respondents will be presented with images of products that are related and unrelated to alcohol. The time limit for each image is 10 seconds. If the respondent does not select any choice within the given time, it will be considered unanswered and the image will be automatically changed to the next one. There are 3 subsections as follows:

Subsection 1: Brand logo perception

In this subsection, the question "*After seeing this image, do you think of alcoholic beverage?*" appears in the beginning. The respondent needs to remember this question. The answers are ✓ for yes and ✕ for no. There are 30 images in total.

Question part:

Please remember this question for this section.<br>**After seeing this image, do you think of alcoholic beverage?**<br>Answers are ✓ for yes and ✕ for no.<br>(You will see the image after pressing the "Start" button.)

Answering part:

Image<br>✕ ✓

Subsection 2: Desire to drink alcohol

In this subsection, the question "*After seeing this image, how much do you want to drink alcohol?*" appears in the beginning. The respondent needs to remember this question. The answers are 0–4, when 0 = do not want at all, 1 = do not want, 2 = neutral, 3 = want to drink and 4 = want to drink very much. There are 10 images in total.

Question part:

Please remember this question for this section.<br>**After seeing this image, how much do you want to drink alcohol?**<br>Answers are 0–4, when 0 = do not want at all, 1 = do not want, 2 = neutral, 3 = want to drink and 4 = want to drink very much<br>(You will see the image after pressing the "Start" button.)<br>Start

Answering part:

Image
0 1 2 3 4

Subsection 3: Frequency

In this subsection, the question "*How often do you see this image?*" appears in the beginning. The respondent needs to remember this question. The answers are everyday (7 days per week), almost every day (5-6 days per week), every other day (3-4 days per week), every week (1-2 days per week), every month (5-6 days per month) and once in a while (Less than once a month). There are 10 images in total.

Question part:

Please remember this question for this section.<br>**How often do you see this image?**<br>Answers are everyday (7 days per week), almost every day (5-6 days per week), every other day (3-4 days per week), every week (1-2 days per week), every month (5-6 days per month) and once in a while (Less than once a month)<br>(You will see the image after pressing the "Start" button.)<br>Start

Answering part:

Image<br>a. Everyday (7 days per week)<br>b. Almost every day (5-6 days per week)<br>c. Every other day (3-4 days per week)<br>d. Every week (1-2 days per week)<br>e. Every month (5-6 days per month)<br>f. Once in a while (Less than once a month)

Section 3: the Alcohol, Smoking, and Substance Involvement Screening Test (ASSIST)

1. In your life, have you ever used alcohol? (If yes, continue the question. If no, continue to the next section.)
    a. Yes
    b. No
2. In the past three months, how often have you used alcohol?
    a. Never
    b. Once or twice
    c. Monthly
    d. Weekly
    e. Daily or almost daily
3. During the past three months, how often have you had a strong desire or urge to use alcohol?
    a. Never
    b. Once or twice
    c. Monthly
    d. Weekly
    e. Daily or almost daily
4. During the past three months, how often has your use of alcohol led to health, social, legal or financial problems?
    a. Never
    b. Once or twice
    c. Monthly
    d. Weekly
    e. Daily or almost daily
5. During the past three months, how often have you failed to do what was normally expected of you because of your use of alcohol?
    a. Never
    b. Once or twice
    c. Monthly
    d. Weekly
    e. Daily or almost daily
6. Has a friend or relative or anyone else overexpressed concern about your use of alcohol?
    a. No, never
    b. Yes, in the past three months
    c. Yes, but not in the past three months
7. Have you ever tried and failed to control, cut down or stop using alcohol?
    a. No, never
    b. Yes, in the past 3 months
    c. Yes, but not in the past three months

Section 4: Personal Information

1. Telephone number (For participants who wish to participate in the prize draw please filled in the phone number. The prizes are 200 baht worth of Starbucks® gift cards for 60 participants. For further information please refer to the participant information sheet.)เพศ
2. Sex
    a. Male
    b. Female
3. Age (years old)
4. Current address
   Province
   District
   Subdistrict
5. Education level
    a. Did not receive formal education
    b. Primary school
    c. Secondary school
    d. Diploma
    e. Bachelor's degree
    f. Higher education
    g. Others
6. Occupation
    a. Agriculture
    b. Business
    c. Private sector employee
    d. Government sector
    e. Retired
    f. Student
    g. Job hunting
    h. Others (please specify)
7. Monthly household income
    a. Less than 5,000 baht/month
    b. 5,000-10,000 baht/month
    c. 10,001-20,000 baht/month
    d. More than 20,000 baht/month

Section 5: Others

1. Do you think that non-alcoholic products of alcoholic brands can influence the sales of alcoholic products?
    a. Yes, I do.
    b. No, I do not.
2. Do you think that alcoholic companies' non-alcoholic products or their related media or advertisement can influence your purchase decision or make you purchase alcoholic products?
    a. Yes, I do.
    b. No, I do not.
3. How easy was it to use this survey application?
    a. Easy
    b. Moderate
    c. Difficult
    d. Very difficult
4. How was the number of questions in this questionnaire?
    a. Too few questions
    b. Adequate
    c. Too many questions
5. Opinion or suggestion for the questionnaire/survey application
